# Multiple evanescent white dot syndrome following BNT162b2 mRNA COVID-19 vaccination

**DOI:** 10.1016/j.ajoc.2022.101532

**Published:** 2022-04-10

**Authors:** Eriko Yasuda, Wataru Matsumiya, Yoshifumi Maeda, Sentaro Kusuhara, Quan Dong Nguyen, Makoto Nakamura, Rumiko Hara

**Affiliations:** a Department of Ophthalmology, Kakogawa Central City Hospital, Kakogawa, Japan; b Department of Surgery, Division of Ophthalmology, Kobe University Graduate School of Medicine, Kobe, Japan; c Spencer Center for Vision Research, Byers Eye Institute, Stanford University, Palo Alto, CA, USA

**Keywords:** Multiple evanescent white dot syndrome, MEWDS, Uveitis, COVID-19 vaccine, Vaccination

## Abstract

**Purpose:**

To report a case with multiple evanescent white dot syndrome (MEWDS) following BNT162b2 mRNA COVID-19 vaccination.

**Observations:**

Case: A 67-year-old Japanese female presented with central visual field loss and photopsia in the right eye (OD) for 5 days. She was complaining blurred vision with bright spots in vision in OD, but denied any ocular symptoms in left eye (OS). She had received the second dose of BNT162b2 mRNA COVID-19 vaccine (Pfizer-BioNTech) one day before the onset of visual symptoms; flu-like symptoms such as mild fever and general fatigue also developed along with ocular symptoms such as decreased vision and hypersensitivity to light in OD following the second COVID-19 vaccine. The first dose of vaccine was administrated followed three weeks later by the second dose and was not associated with any ocular or systemic symptoms besides mild pain at the injection site. She had not been followed by any ophthalmologist before the initial visit. At the initial visit, best corrected visual acuity (BCVA) in decimal points was 0.2 in OD and 1.0 in OS. Ophthalmic examination showed multifocal white dots in the posterior retina with moderate vitritis (1+ haze and 2+ cells) in OD. Multimodal imaging in OD showed diffuse disruption of ellipsoid zone with variable punctate hyperreflective lesions at macula on optical coherence tomography, punctate hyperfluorescence in a wreath-like pattern and late staining on fluorescein angiography, and multiple hypofluorescent spots of various sizes in the late phases on indocyanine green angiography. Both multiple hypofluorescent spots and scattered hyperfluorescent spots corresponding to white dots in OD were also seen on fundus autofluorescence. Her laboratory and systemic evaluations were negative for syphilis, tuberculosis, or toxoplasma, and selected autoimmune diseases like sarcoidosis, Behcet's disease, rheumatoid arthritis, and systemic lupus erythematosus. No active intraocular inflammation or abnormality were seen in OS. One week later, the multifocal white dots disappeared in OD, and were almost invisible on fundus photography. At that time, multifocal electroretinogram showed decreased response with low amplitude density across the entire field in OD. The BCVA in OD spontaneously improved to 0.8 without any treatment. Collectively, these clinical course and findings were suggestive of a diagnosis of MEWDS after mRNA COVID-19 vaccination.

**Conclusions and importance:**

In this present case, BNT162b2 mRNA COVID-19 vaccination might have been associated with MEWDS-like entity with vision loss. It is important for physicians to monitor the ocular status carefully in patients with visual disturbance after COVID-19 vaccination.

## 1 Introduction

Since the pandemic of Coronavirus disease 2019 (COVID-19) caused by coronavirus-2 (SARS-CoV-2) was first reported in late 2019, the global world has faced new challenges. As various COVID-19 vaccines have shown their efficacy in clinical trials, the World Health Organization (WHO) has also expected that safe and effective COVID-19 vaccine can be a game-changer.^1^^,^^2^ However, these expectations has placed tremendous pressure on vaccine-makers to supply vaccine as soon as possible and to allow them to accept safety testing to be performed in less than one year, which is significantly shorter than the 12–15 years typically required for the commercialization of a vaccine.^3^ Therefore, COVID-19 vaccine may have potential pitfalls secondary to the lack of formal phase II and III clinical trials due to acute high demand of supply. There is no substitute currently available for long-term human clinical trials to ensure long-term human safety.^4^ Thus, physicians need to monitor the safety status of COVID-19 vaccination and ask the patient about the history of COVID-19 vaccination as necessary when the patients present with atypical symptoms or develop adverse events such as visual disturbance.

In fact, reports of vaccine-associated uveitis have been accumulated as local adverse reaction of vaccines, although it is not common.^5^ Cases of multiple evanescent white dot syndrome (MEWDS) following various vaccination have been reported.^6^ According to the report by Ng et al. in their review, the median time from vaccination was 14 days with a range from 1 day to 30 days.

Recently, 21 cases with anterior uveitis and two cases with MEWDS after mRNA COVID-19 vaccination have been reported.^7^ Since posterior uveitis such as MEWDS can cause visual impairment, it is important to evaluate in detail for ocular inflammation after COVID-19 vaccination as possible adverse effect related to COVID-19 vaccine. We herein describe a case of MEWDS with significant vitritis following the second dose of BNT162b2 mRNA COVID-19 vaccine (Pfizer-BioNTech).

## 2 Case report

A 67-year-old Japanese female presented with central visual field loss and photopsia in the right eye (OD) for 5 days. She was complaining blurred vision with bright spots in vision in OD, but denied any visual symptoms in her left eye (OS). She had received the second dose of BNT162b2 mRNA COVID-19 vaccine (Pfizer-BioNTech) one day before the onset of visual loss; flu-like symptoms such as mild fever and general fatigue along with visual symptoms developed following the second COVID-19 vaccine, which was administered three weeks after the first dose which did not bring any ocular or systemic symptoms besides mild pain at injection site. Since the inception of the pandemic, she had gotten no experience of COVID-19 symptoms and had not tested for SARS-CoV-2 by real-time polymerase chain reaction (RT-PCR). She had not been followed by any ophthalmologist before initial visit. Past medical, drug, and family histories were unremarkable.

**Fig. 1:**
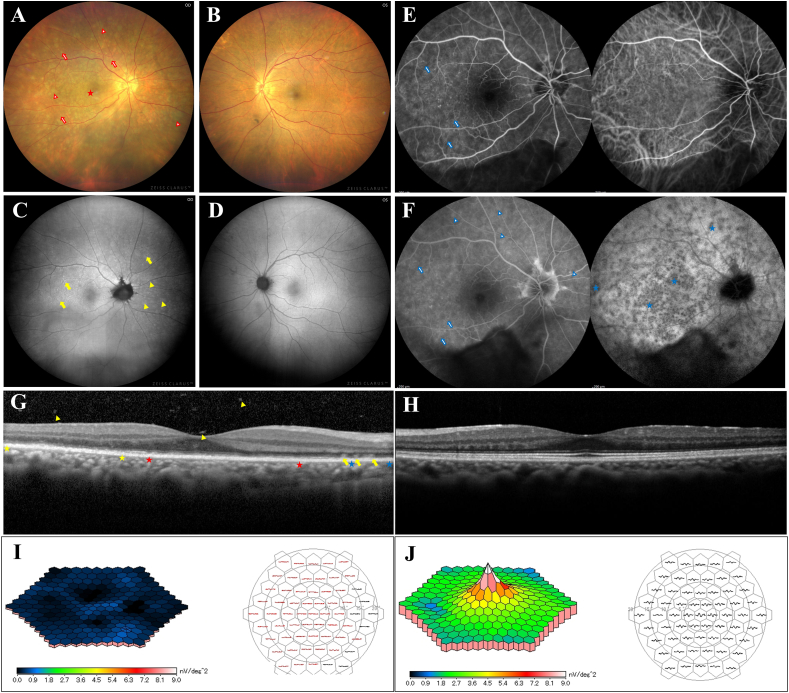
Multimodal imaging including fundus photo (FP) (A,B), fundus autofluorescence (FAF) (C,D), fluorescein angiography (FA), indocyanine green angiography (ICGA) (E,F), and optical coherence tomography (OCT) (G, H) at initial visit and multifocal electroretinography (ERG) at one week (I,J). On FP, concentrated multifocal white small dots (arrows) and larger spots (arrow heads) with sparing fovea itself (star), around posterior pole and optic disc were shown in the right eye (A) and there was unremarkable finding in the left eye besides artifacts looking small dots(B). On FAF, there were mixed multiple hypofluorescent spots surrounded by small hyperfluorescent circles (arrows) and scattered hyperfluorescent lesions (arrow heads) corresponding to white dots in the right eye (C) and no abnormal fluorescence in the left eye (D). On FA, the early-middle phase at 1 minute (E left) and the late phase at 20 minutes (F left), punctate hyperfluorescence in a wreath-like pattern (arrows) and late multiple staining spots (arrow heads) were seen in the right eye. On ICGA, in the late phase at 20 minutes (F right), multiple hypofluorescence spots (stars) with various sizes were prominently seen. The blocked fluorescence due to vitreous opacities were seen on the bottom of images on FA and ICGA (E,F). On horizontal OCT scan through the fovea, there were variable hyperreflective lesions spots in the outer retina (arrows), diffuse disruption in the ellipsoid zone (between same color stars), and some hyperreflective dots (arrow heads) in the vitreous were seen in the right eye (G) and normal structure at macula in the left eye (H). Multifocal ERGs showed decreased retinal response with low-amplitude density at the entire field in the right eye (I) and normal retinal response amplitude density in the left eye (J). . (For interpretation of the references to color in this figure legend, the reader is referred to the Web version of this article.)

On ophthalmic examination at the initial visit, her best corrected visual acuity (BCVA) shown as decimal points by Landolt C test (Snellen equivalence) was 0.2 (20/100) OD and 1.0 (20/20) OS. Intraocular pressure was 13 mmHg OD and 14 mmHg OS. Pupils were equally round and reactive to light and accommodation and extraocular motility was normal. On slit-lamp examination, anterior segments were normal except for bilateral mild cataract. However, moderate vitritis (1+ haze and 2+ cells) with some opacities in anterior vitreous and scattered vitreous opacities including small white balls in the mid to posterior vitreous were detected in OD. On fundus examination, multifocal small white dots at the deep retinal layers were seen around the posterior pole and optic disc (Fig. 1A). Anterior and posterior segments of OS were unremarkable. On fundus autofluorescence (FAF), there were mixed multiple hypofluorescent spots surrounded by small hyperfluorescent circles and scattered hyperfluorescent lesions, which were concentrated around optic disc and posterior pole in OD (Fig. 1C). Fluorescein angiography (FA) showed early punctate hyperfluorescence in a wreath-like pattern with late staining; indocyanine green angiography (ICGA) indicated multiple hypofluorescent spots of various sizes especially in the late phases (Fig. 1E and F). Optical coherence tomography (OCT) showed punctate hyperreflective lesions of variable sizes in the outer retina and diffuse disruption in the ellipsoid zone (EZ) at macula. (Fig. 1G). Goldmann visual field test showed a central scotoma and paracentral islands of sensitivity loss in OD.

**Fig. 2:**
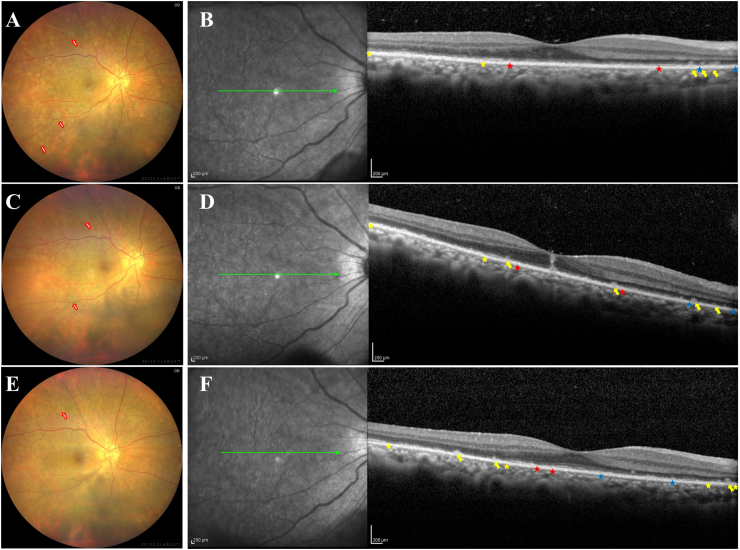
Fundus photo (FP) and optical coherence tomography (OCT) at initial visit (A,B), one week (C,D), and two weeks (E,F). On FP, multifocal variable punctate (arrows) white spots which were prominently seen at initial visit (A) had started to disappear at one week and two weeks (B, C). On horizontal OCT scan through the fovea, although hyperreflective lesions in the outer retina (arrows) mildly increased at one week (D) compared to initial visit (B), there was no significant difference about diffuse disruption in the ellipsoid zone (between same color stars) between at initial visit (B) and at one week (D). However, OCT at 2 weeks showed recovering ellipsoid zone with less hyperreflective lesions (arrows) in the outer retina, which corresponded to improving visual acuity in the right eye. The follow-up OCT scans (D and F) were obtained via the Auto Rescan™ function of Heidelberg Spectral (Heidelberg Engineering, Heidelberg, Germany) by setting the baseline scan (A) as a reference scan. . (For interpretation of the references to color in this figure legend, the reader is referred to the Web version of this article.)

One week later the multifocal white dots had disappeared in OD and were almost invisible on fundus photography, although RPE color changes at posterior pole along with active vitritis were seen (Fig. 2C and D). Her BCVA was 0.4 (20/50) OD and 1.2 (20/16) OS at that time. Multifocal ERGs showed decreased retinal response with low-amplitude density over the entire field in OD and normal retinal response amplitude density in OS (Fig. 1I and J). Such ERG finding was consistent with the central scotoma on Goldman visual field test and suboptimal vision in OD. Her laboratory evaluations were negative for syphilis, tuberculosis, or toxoplasma, and selected autoimmune diseases like sarcoidosis. No active intraocular inflammation was seen in OS. Two weeks after the initial presentation, her BCVA OD spontaneously improved to 0.8 (20/25) without any treatment. In addition, OCT findings demonstrated improved EZ and less punctate hyperreflective lesions in OD (Fig. 2E and F). Collectively, based on the clinical course and findings, a diagnosis of MEWDS after mRNA COVID-19 vaccination was made.

## 3 Discussion

We have presented a case of a 67-year-old Japanese female who developed multifocal white dots concentrated around posterior pole and optic disc with moderate vitritis following the second BNT162b2 mRNA COVID-19 vaccination. Given the negative results of infectious etiologies including syphilis, tuberculosis, and toxoplasma, the differential diagnoses included MEWDS, acute posterior multifocal placoid pigment epitheliopathy (AMPPE), multifocal choroiditis (MFC), and birdshot chorioretinopathy, among others. Multimodal imaging of the affected eye showed typical findings as MEWDS in the present case. Wide angle fundus photo showed time-course changes of disappearance of multifocal white dots in the retina of OD (Fig. 2A, 2C, 2E). Although scattered white dots were seen at the initial visit, those dots started to disappear one week later. However, moderate vitritis and RPE changes at macula remained at that time. Diffuse disruption of the EZ at the macula and punctate hyperreflective lesions of variable size in the outer retina on OCT were consistent with MEWDS. Classic signs of MEWDS in angiography, early punctate hyperfluorescence in a wreath-like pattern, late staining on FA, and late hypofluorescent dots and spots on ICGA in OD corresponded to the grey-white dots. The findings on ocular imaging indicated that the main inflammation was located on outer retina and RPE/Bruch membrane.

Despite the maculopathy with moderate vitritis which resulted in suboptimal visual acuity, given the findings on multimodal imaging and clinical characteristics, including spontaneous resolution, unilateral ocular inflammation and female gender, which were common in MEWDS, we made the diagnosis of MEWDS for our index patient. Although most cases with MEWDS present with an infection prior to the onset of ocular symptoms, the present case had not had flu-like symptoms but had received the second dose of BNT162b2 mRNA COVID-19 vaccine one day before the onset of ocular symptoms.^8^ Therefore, it is possible that the COVID-19 vaccination-induced ocular inflammation manifested as MEWDS in the present case.

Characteristics of patients with post-vaccine MEWDS were as follows: healthy (77.8%), young to middle age (median 33 years; mean 31.7years; range 16–53 years), women (66.7%), Caucasian (44%).^6^ Recently, several cases of ocular adverse events have also been reported after COVID-19 vaccination.^9^ Among them, acute idiopathic maculopathy, acute macular neuroretinopathy (AMN), and Vogt-Koyanagi-Harada disease (VKH) besides MEWDS have been reported as posterior ocular manifestation.^7^^,^^10^, ^11^, ^12^, ^13^, ^14^, ^15^

Globally, people have benefited tremendously from vaccination since the smallpox vaccine was developed by Edward Jenner in 1796,^16^^,^^17^ although it is known that vaccines may also have a variety of potential side effects. According to the reports about vaccine-associated uveitis by Benage et al., 289 cases of vaccine-associated uveitis were collected from the National Registry of Drug-Induced Ocular Side Effects (www.eyedrugregistry.com), the WHO Monitoring Centre (Uppsala, Sweden), and the FDA spontaneous reporting system during a 26- year period from 1984 and 2014.^5^ In the report, the most common vaccine associated with uveitis was hepatitis B vaccine (115 cases), followed by human papillomavirus vaccine (44 cases), influenza vaccine (28 cases), Bacille Calmette-Guerin (BCG) vaccine (21 cases), and measles-mumps-rubella (MMR) vaccine and varicella vaccine (13 cases each). The median number of days between vaccination and onset of uveitis was 16 days, ranging from one day to six years.

Thus far, several mechanisms about vaccine-associated uveitis have been suggested, although the pathogenesis has not been elucidated in most patients. One of the possible mechanisms was that ocular inflammation following vaccine could be directly induced by live, attenuated vaccines, such as MMR vaccine, because there might still be active, viral strain in the vaccines. It was reported that varicella zoster virus was detected from the eye with active inflammation after attenuated varicella vaccine, although that is rare.^18^

As another mechanism, it was proposed that post-vaccinated ocular inflammation could be led by the similarity between adjuvants of vaccine and uveal self-antigen as antibody-mediated hypersensitivity reactions. In the case of the hepatitis A vaccine, aluminum salt as the adjuvant used in the inactivated vaccine was reported as a possible cause of vaccine-associated uveitis.^19^, ^20^, ^21^

When a patient has a genetic background which includes a family or personal history of autoimmune disease, the patient can be regarded as autoinflammatory, and is susceptible to autoimmune conditions induced by adjuvants (ASIA), known collectively as Shoenfeld syndrome.^22^ However, the index patient did not have any apparent genetic predisposition to autoimmune diseases.

BNT162b2 mRNA COVID-19 vaccine with encoding the SARS-CoV-2 full-length spike was made to target S protein, and then it has been documented effective in reducing COVID-19.^23^

In terms of side effects after vaccination, according to phase 3 clinical trials of the BNT162b2 vaccine, the most common events after the first dose were injection-site pain (71–83%), fatigue (34–47%), and headache (25–42%).^23^ It was reported that systemic reactogenicity was more common and severe after the second dose than after the first dose, although local reactogenicity was similar after the two doses. Uveitis was not reported as an adverse event in registration studies; however, a case of anterior uveitis in a 23-year-old male, with onset 14 days after the second dose of BNT162b2 COVID-19 vaccine has been reported recently.^9^

In terms of the concept of vaccine, mRNA vaccine is quite different from the inactivated vaccine and live attenuated vaccines and does not contain aluminum salts as adjuvants in its formulation.^9^ Therefore, newer theory about vaccine-associated inflammation has been proposed instead of the aluminum salts adjuvant hypothesis. In the Pfizer-BioNTech vaccine and the Moderna vaccine, mRNA is encapsulated in two novel lipid nanoparticles, one of which is polyethylene glycol (PEG). The possibility that PEG can induce allergic reactions are reported in the literature, which showed IgE antibodies in patients with a history of PEG induced anaphylaxis^24^ and PEG induced complement activation.^25^ Although the PEG allergy is uncommon, PEG may be a potential trigger to immunoreaction after mRNA COVID-19 vaccination. Recently, Rabinovitch et al., reported 19 cases with anterior uveitis including two cases of bilateral inflammation and two cases with unilateral MEWDS following BNT162b2 mRNA COVID-19 vaccination. According to the report, most cases of COVID-19 vaccine-associated uveitis had unilateral inflammation like the present case.^7^ In contrast to the cases with unilateral MEWDS associated with mRNA COVID-19 vaccination that have been described, our case showed relatively more severe ocular inflammation. Her visual acuity decreased to 0.2 (20/100) OD in the present case, which was quite worse than 20/32 in both cases with MEWDS in the recent report. Although they did not show any sign of ocular inflammation like most cases with MEWDS except retinal changes, the present case had moderate vitritis with scattered vitreous opacities with small white balls in OD. In addition, the present case had a shorter time period interval between vaccination and the onset of symptoms, which was 1 day after the second dose of vaccination, compared to 5 days and 30 days in the two cases with MEWDS. The index patient was 67-year-old, which was older compared to middle age, younger-than-50 years of age in most cases of MEWDS.^26^ Although the present case showed visual recovery without any treatment as commonly described, the case indicates that MEWDS following mRNA COVID-19 vaccination may have atypical demographic characteristics or clinical findings.

Among vaccine-associated uveitis, several cases with vaccination-associated MEWDS have been also reported, which were presented after the vaccination of rabies, human papillomavirus, hepatitis A, hepatitis B, meningococcal, Yellow fever, and influenza.^6^ Although the exact pathogenesis of MEWDS is yet to be fully elucidated since multiple evanescent white dot syndrome (MEWDS) was first described in 1984, most recent hypotheses suggest an immune-mediated mechanism occurring at either the outer retina,^27^^,^^28^ the choriocapillaris/inner choroid,^29^ or both.

The onset of present case was one day after the second dose of COVID-19 vaccination. According to the previous reports, Rabinovitch et al., reported uveitis developed in 15 eyes (13 patients) out of 21 patients after the second vaccine dose: 13 anterior uveitis and two MEWDS cases. Bolletta E. et al. also reported one case of MEWDS that developed after thesecond dose of vaccination. In their report, over 50% of uveitis or other ocular complications following COVID-19 vaccination were noticed after the second dose. It has been mentioned by Centers for Disease Control and Prevention (CDC) that adverse reactions (such as fever, chills, tiredness, and headache) throughout the body are more common after the second dose of the BNT162b2 COVID-19 vaccine. One report showed that in the majority of cases, the major complaint is a combination of fever, headache, myalgia, and general malaise, affecting almost 60% of recipients after the second dose of the vaccines as well.^30^ Therefore, uveitis after the second dose might be possible like the present case.

It was reported that most of the symptoms could be as a result of much production of a cytokine that is crucial in potentiating early stages of the immune response like type I interferon (IFN–I) by macrophages and dendritic cells (DC).^31^ Discussions in some reports were also focusing on a role of vaccine-induced IFN-1 between BNT162b2 mRNA COVID-19 vaccination and uveitis following vaccination. The mRNA vaccine-induced IFN-I through RNA sensors including RIG-I promotes differentiation of CD4^+^ and CD8^+^ effector T cells associated with inflammatory and cytotoxic mediators, and CD4^+^ helper T cells promoting B cell differentiation into plasma cells with the ability of antibody-secretion.^7^^,^^11^

IFN-I -producing DCs and other cells that have taken up the vaccine-derived nucleic acids encoding the S protein can deliver both an antigenic and inflammatory signal to T cells in lymph nodes draining the injection site. This process activates S protein-specific T cells and induces adaptive immunity against SARS-CoV-2.^32^ Moreover, IFN-1 has been shown to amplify T cell memory and promote B cell differentiation and survival, suggesting vaccine-associated inflammation in the booster can further promote generation and perpetuation of long-term immunological memory.^32^ This secondary enhancement of the inflammatory adverse response due to the second dose of mRNA vaccine which require two doses spaced 3–4 weeks apart to promote optimal protection can be explained by immunological memory formation of trained immunity by effector T cell via IFN-1 production and/or by activation of memory T cells and B cells generated from the initial injection.^33^ In addition, it was reported that the activation of IFN response via RIG-I induced expression of cell death effectors and caused barrier function loss in RPE cells.^34^ Therefore, this mechanism may explain why MEWDS whose inflammation was located at the outer retina, the choriocapillaris/inner choroid, or both could develop after the second dose.

Therefore, BNT162b2 mRNA COVID-19 vaccination could play a role of an immunologic trigger for the development of ocular inflammatory disease manifested as MEWDS, although it still remains possible that ocular inflammation following vaccination coincidentally occurred. Fortunately, BCVA in OD in the present case spontaneously improved to 0.8 without any treatment. However, it is hoped that our report would alert physicians to the potential risk of ocular inflammation which might temporarily cause vision loss and MEWDS-like entity, following COVID-19 vaccination.

## Patient consent

Consents to publish have been obtained from the patients.

## Funding

NA.

## Authorship

All authors attest that they met the current ICMJE criteria.

## Declaration of competing interest

The authors declare that there are no conflicts of interest related to this manuscript.
